# Evaluation of Color Difference Models for Wide Color Gamut and High Dynamic Range

**DOI:** 10.3390/jimaging10120317

**Published:** 2024-12-10

**Authors:** Olga Basova, Sergey Gladilin, Vladislav Kokhan, Mikhalina Kharkevich, Anastasia Sarycheva, Ivan Konovalenko, Mikhail Chobanu, Ilya Nikolaev

**Affiliations:** 1 Federal Research Center “Computer Science and Control” of the Russian Academy of Sciences, 119333 Moscow, Russiakonovalenko@vis.iitp.ru (I.K.); i.p.nikolaev@iitp.ru (I.N.); 2 Smart Engines Service LLC., 117312 Moscow, Russia; 3 Institute for Information Transmission Problems of the Russian Academy of Sciences, 127051 Moscow, Russia; 4 Department of Computing Machines, Systems and Networks, Institute of Information Technologies and Computer Science, National Research University “Moscow Power Engineering Institute”, 111250 Moscow, Russia

**Keywords:** color difference model, color model evaluation, color difference dataset, strict substitution, gray-scale method, wide color gamut, high dynamic range

## Abstract

Color difference models (CDMs) are essential for accurate color reproduction in image processing. While CDMs aim to reflect perceived color differences (CDs) from psychophysical data, they remain largely untested in wide color gamut (WCG) and high dynamic range (HDR) contexts, which are underrepresented in current datasets. This gap highlights the need to validate CDMs across WCG and HDR. Moreover, the non-geodesic structure of perceptual color space necessitates datasets covering CDs of various magnitudes, while most existing datasets emphasize only small and threshold CDs. To address this, we collected a new dataset encompassing a broad range of CDs in WCG and HDR contexts and developed a novel CDM fitted to these data. Benchmarking various CDMs using STRESS and significant error fractions on both new and established datasets reveals that CAM16-UCS with power correction is the most versatile model, delivering strong average performance across WCG colors up to 1611 cd/m^2^. However, even the best CDM fails to achieve the desired accuracy limits and yields significant errors. CAM16-UCS, though promising, requires further refinement, particularly in its power correction component to better capture the non-geodesic structure of perceptual color space.

## 1. Introduction

With rapid advancements in high dynamic range (HDR) display technology—now achieving luminance levels exceeding 1000 cd/m^2^ in accordance with new HDR standards [1,2], which also incorporate wide color gamut (WCG) defined in the earlier BT.2020 standard [3]—tools for precise color reproduction assessment have become essential. Color difference models (CDMs) play a crucial role by estimating perceived color differences (CDs) between colors. Mathematically, a CDM can be expressed as a function $F(c_{1},c_{2},p)\to d$, where *d* is the perceived difference between colors $c_{1}$ and $c_{2}$, and *p* represents the observation parameters. A basic CDM approach uses the Euclidean distance in a uniform color space (UCS), described as $F\left(c_{1},c_{2},p\right)=||T\left(c_{1},p\right)-T\left(c_{2},p\right){\left|\right|}_{2}$, where *T* is the transformation from the colorimetry color space to the particular UCS. A UCS can be used not only as a CDM but also as a space for performing color transformations. It has been shown that the performance of image processing algorithms significantly depends on the chosen CS or CDM, as demonstrated in applications such as tone mapping [4], color correction [5], and image recognition [6].

CDMs are developed and validated using datasets comprising color pairs and their perceived differences, as determined through psychophysical experiments. While various CDMs have been proposed for HDR contexts [7,8,9,10,11], HDR- and WCG-specific experimental data to evaluate these models are still sparse. Existing datasets, often collected based on textile and printed samples [12,13,14,15], lack WCG coverage, and recent monitor-based datasets are confined to luminance levels up to 300 cd/m^2^ [16,17]. Thus, dedicated experimental setups are necessary to acquire data covering both HDR and WCG ranges.

There are various types of experiments to assess CDs with different magnitudes: threshold CD (TCD) or just noticeable difference (JND), small CD (SCD), and large CD (LCD, exceeding 5–8 JNDs). Measuring CDs across these ranges is critical due to the non-geodesic nature of perceptual color space, where the cumulative JNDs along the shortest path (geodesic) can surpass the direct CD between endpoints [18,19,20]. This discrepancy challenges traditional UCS models using Euclidean metrics for varying CD magnitudes. Consequently, UCSs specifically designed for LCD or SCD applications based on the CIECAM02 model [21] have been developed, and a generalized approach using power functions (with exponents <1) has been applied to current UCS models [22], including the state-of-the-art CAM16-UCS [23].

A recent review by Luo et al. [24] evaluated psychophysical datasets on CDs from over 20 studies, benchmarking CDMs across these datasets. For decades, such datasets have played a central role in the iterative development of CDMs, forming the foundation for refining gold-standard models widely used in color evaluation. This progress has, in turn, driven advancements in applied algorithms. While HDR datasets [17] (up to 1128 cd/m^2^) and WCG datasets [25] (covering the BT.2020 gamut) exist, the review acknowledges a significant gap: the absence of datasets encompassing both HDR and WCG. Additionally, existing HDR and WCG datasets are limited in their coverage of large CDs. Large CDs are particularly important in display manufacturing, where defective pixels significantly affect perceived color [26]. Addressing these gaps is critical for further advancing UCS development, necessitating new datasets that cover WCG, HDR, and large CD ranges. To this end, we obtained a new color difference dataset spanning threshold, small, and large CDs in HDR and WCG. Using this dataset, we developed an analytically fitted CDM and benchmarked several CDMs with STRESS and significant error fraction metrics across both new and established datasets, providing a robust foundation for future UCS development.

## 2. Materials and Methods

### 2.1. Developed Experiment Setup

To obtain psychophysical data across HDR and WCG color spaces at various color difference (CD) scales, we developed a custom experimental setup centered around a purpose-built stimulus generator. Since no commercially available device met the required HDR and WCG specifications, we collaborated with Visionica Ltd. (Moscow, Russia) (http://www.visionica.biz/, accessed 8 November 2024) to design a stimulus generator, named VCD. VCD provides four independent stimuli, each comprising several dozen LEDs per primary color, ensuring both high luminance (up to 5000 cd/m^2^) and uniformity of the light field.

**Figure 1:**
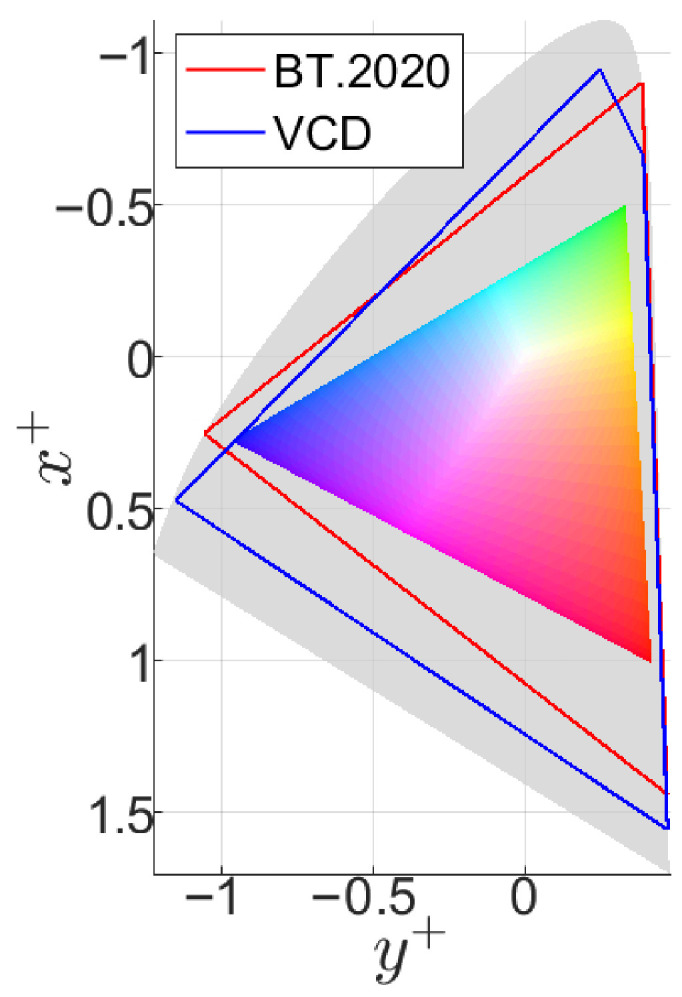
The VCD gamut (blue), BT.2020 (red), human visual gamut (gray), and sRGB gamut (colored) shown on the proLab chromaticity diagram.

Our goal was to cover as much of the BT.2020 gamut as possible using the VCD. While the BT.2020 [1] gamut triangle is theoretically defined, its primaries, located on the spectral locus, can only be practically achieved using lasers. With LEDs, it is feasible to achieve two out of three primaries with adequate luminance levels, but achieving a high-saturation green LED with sufficient luminance remains a challenge. This issue, known as the “green gap” [27], arises from the physical limitations of current LED technology. To address this, we incorporated two green LEDs with different spectral power distributions, resulting in a total of four LEDs in the VCD. This configuration expanded the device’s color gamut (see Figure 1). Adding more LEDs would provide only marginal gains in gamut coverage while significantly complicating the device design.

**Figure 2:**
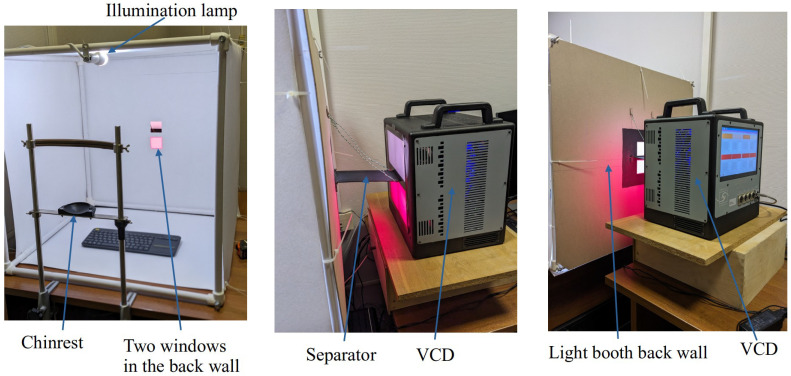
Front and side views of the experiment setup.

The full experimental setup included a light booth with two rear windows (for different scenarios), an illuminator, and the custom stimulus generator discussed above. Figure 2 shows views of the setup.

**Figure 3:**
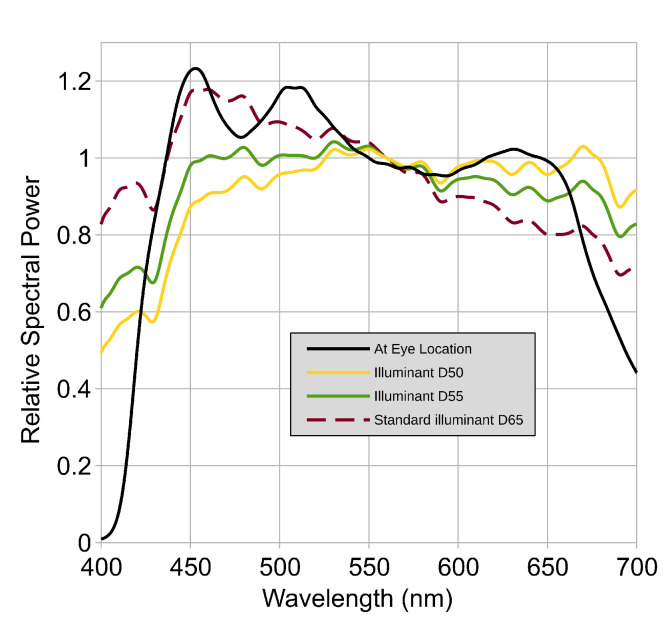
Spectral power distributions of the luminance inside the booth compared to CIE D50, D55, and D65 illuminants.

The light booth, a 70 × 70 × 70 cm cube with five white paper walls, was top-lit by a Remez 7W 5700K LED lamp (color rendering index: 98.5 and correlated color temperature: 5553 K), providing 100 cd/m^2^ at the center of the back wall and ensuring uniform lighting. Spectral measurements were conducted using an X-Rite I1Pro spectrophotometer (3.3 nm resolution) with ArgyllCMS software (version 2.1.2) (http://argyllcms.com/, accessed 8 November 2024). Figure 3 shows the spectral power distributions (SPDs) inside the booth alongside CIE D50, D55, and D65 illuminants.

**Figure 4:**
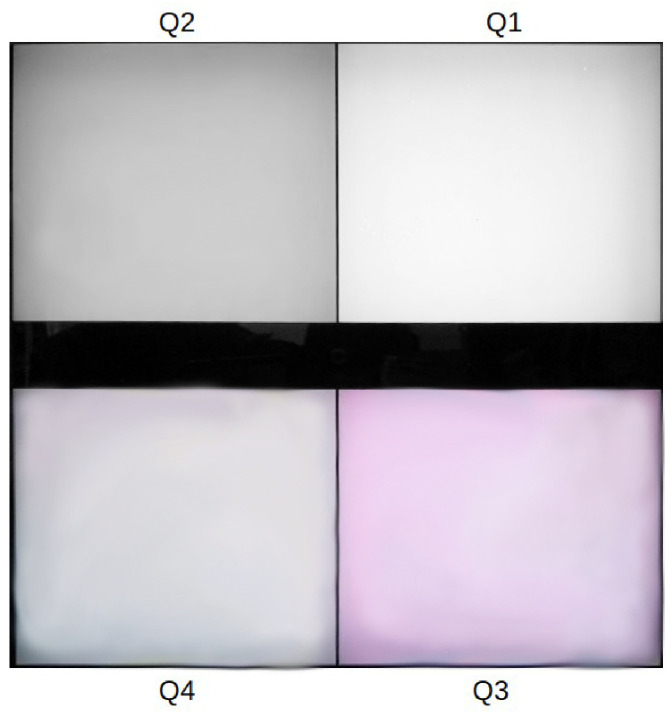
Stimuli setup for SCD experiments. Quadrants: Q1, upper right; Q2, upper left; Q3, lower right; and Q4, lower left.

The VCD generator produced four stimuli (10 × 10 cm quadrants), each controlled independently. A 1 mm vertical separator divided the stimuli horizontally, with a 2 cm space between the upper and lower pairs; see Figure 4. The VCD was positioned 17 cm from the booth’s back wall, and a black separator was placed between viewing windows. Observers sat 80 cm from the windows, ensuring an angular stimulus size of 3.58°.

**Figure 5:**
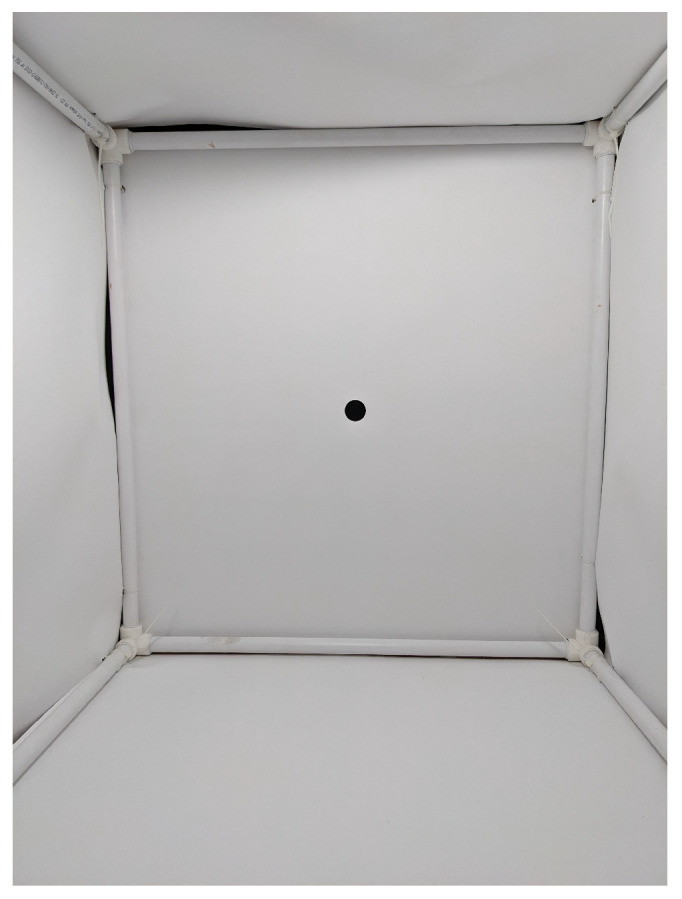
Interior of the light booth for TCD measurements.

For TCD measurements, a 28 mm round hole in the back wall provided a 2° angular stimulus size; see Figure 5.

For SCD and LCD measurements, two 5 × 5 cm rectangular windows separated by 2 cm were used. The chinrest was adjusted to block the observer’s view of gaps between the VCD quadrants.

#### 2.1.1. VCD Stimulus Generator Characterization

The VCD stimulus generator produced four independently controlled stimuli, each in a separate quadrant (Figure 4). Each quadrant featured a board with four primary LEDs (see Section 2.1.2) controlled at 16-bit accuracy, with settings managed remotely by a computer.

Uniformity testing via photographs revealed a 15% luminance drop along the left edge of Q1 near Q2, consistent across quadrants and imperceptible to the human eye. This variation was negligible for SCD and LCD measurements (see Section 2.2.2) but became significant for TCD if colors were compared side by side. Therefore, we employed an alternate procedure for TCD, where colors were flashed alternately within the same quadrant (see Section 2.2.1).

#### 2.1.2. Primaries Spectra

**Figure 6:**
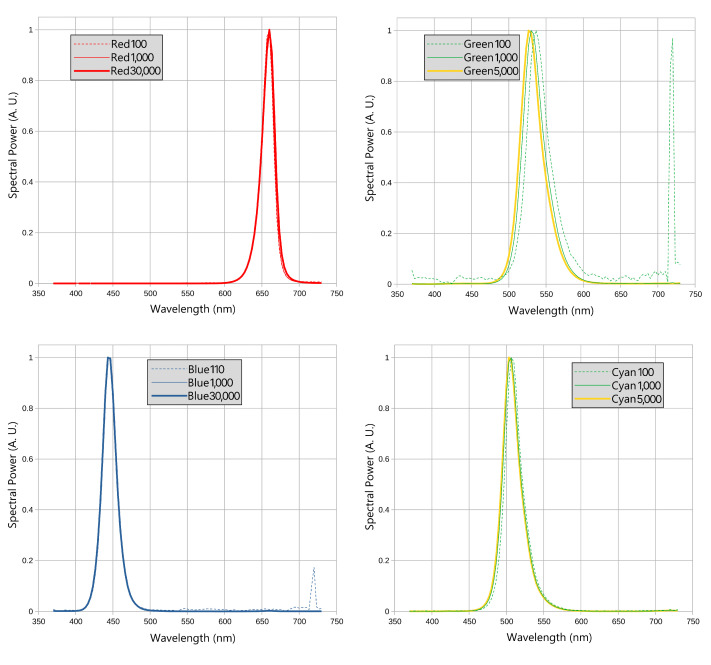
Spectral power distributions of Q1 primaries R, G, B, and C.

**Figure 7:**
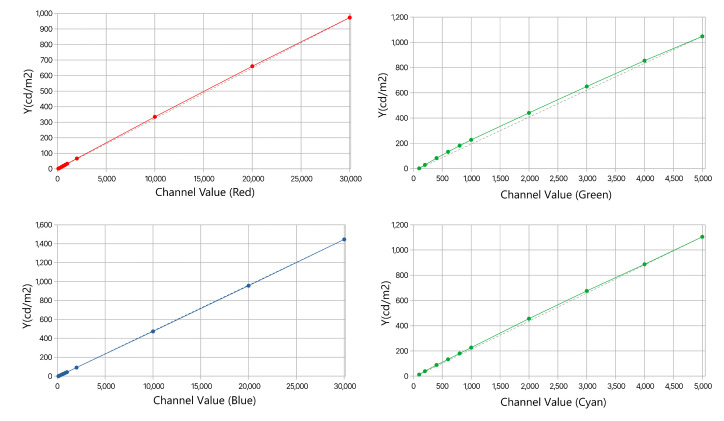
Luminance vs. channel value for Q1 primaries (left to right, top to bottom: R, G, B, and C). Dots illustrate measurements; dashed lines represent ideal linearity; and solid lines, parabolic approximation.

The VCD featured four primary channels—red (R), green (G), blue (B), and cyan (C)—each controlled independently via 16-bit buses, allowing luminance values from 0 to 65,535. Spectral power distributions (SPDs) were measured for various channel values in Q1 (Figure 6), with settings of 30,000 for R and B yielding luminance around 1000 and 1400 cd/m^2^, and 5000 for G and C yielding around 1000 and 1100 cd/m^2^. Minor SPD shifts due to LED heating, most notable in the green channel, were consistent across quadrants and accounted for in the VCD control software. Luminance linearity was assessed by measuring primary outputs at various channel values, approximated by a parabolic function to ensure accuracy (Figure 7).

To verify calibration, we compared the chromaticity coordinates of the target colors that the VCD intended to produce with the actual colors formed, which were measured using an X-Rite I1Pro spectrophotometer in its colorimeter mode. Approximately 40 different xyY values, evenly distributed across the chromaticity diagram at two luminance levels (300 and 1000 cd/m^2^), were tested. Chromaticity errors were calculated as the distance between the measured and target colors on the CIE 1964 xy chromaticity diagram for the 10° observer. The chromaticity error was found to depend on both the y and Y coordinates, ranging from 0.007 (for y < 0.5 at 1000 cd/m^2^) to 0.038 (for y ≥ 0.6 at 300 cd/m^2^), with a mean error of 0.017, which is considered small.

### 2.2. Experimental Procedures

#### 2.2.1. TCD Measurements

TCD measurements were conducted using the “strict substitution” technique [28,29]. In this method, a color center (reference color, Stimulus A) is displayed, and a test color (Stimulus B) is shifted in a chosen direction within the color space away from the color center. Each stimulus is shown for 1.5 s in a periodic, instantaneous alternation.

Observers are given unlimited time to detect any difference; if none is observed, they increase the shift of Stimulus B by pressing a button. The just noticeable difference (JND) is the smallest shift at which the observer perceives a distinction between the two stimuli.

**Figure 8:**
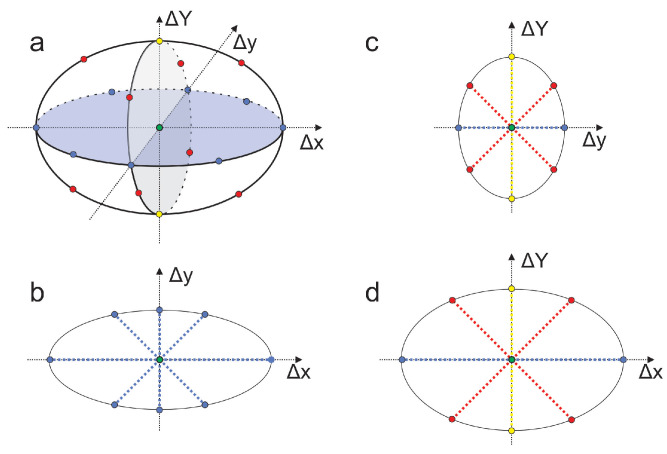
Arrangement of 18 measurement directions around the color center in xyY space. Blue points indicate chromaticity-only shifts (ΔY=0), yellow points show luminance shifts (Δx=0,Δy=0), and red points represent combined chromaticity and luminance shifts. (**a**) 3D view; (**b**) section showing chromaticity-only shifts; (**c**) section with x-coordinate fixed at 0; (**d**) section with y-coordinate fixed.

Stimulus B is shifted in discrete steps along specific directions in CIE xyY space. While a smooth surface would require measurement in numerous directions, which was not feasible, we used 18 directions combining chromaticity and luminance shifts and 8 directions with fixed luminance to approximate this surface (details in Figure 8).

#### 2.2.2. SCD and LCD Measurements

SCD and LCD were measured using the “gray-scale method”, which matches color differences to a gray reference scale [25,30,31,32]. Observers viewed two pairs of colors: a reference gray pair with a known difference (3 JND for SCD, 12 JND for LCD) and a test pair.

The viewed stimuli are shown in Figure 4. The reference gray pair (stimuli Q1 and Q2) had a fixed difference, while the test pair (Q3 and Q4) started at the same color center (Q4). Observers incrementally adjusted Q3 along one of 18 preset directions (Figure 8) until its perceived difference from Q4 matched that of the gray reference pair. Upon matching, the color center and the selected shift were recorded.

The reference pair had chromaticity coordinates corresponding to D65. One sample in the reference pair remained fixed as GS-1, with the other as GS-3 for SCD or GS-5 for LCD measurements (see Table 1). For improved observer’s brightness adaptation, gray-scale luminances were increased 20-fold, with a reference white luminance of 2000 cd/m^2^, as detailed in Table 1, where $L^{*}$ denotes the CIELAB lightness value.

#### 2.2.3. Operating Conditions

Human color perception is strongly influenced by viewing conditions. For instance, the perceived magnitude of color differences depends on the chromatic adaptation of the human visual system to dominant illumination [33,34]. Chromatic adaptation transforms (CATs) describe these dependencies [23,35] and are often integrated into UCSs [23,36]. Developing such models requires the collection of specialized datasets [37,38]. However, it is not feasible to cover all possible scenarios within a single dataset, as this would require sampling a highly multidimensional parameter space. Consequently, different studies typically focus on specific subsets of this space. In our work, we addressed a four-dimensional space comprising three color coordinates and the magnitude of color difference. Nonetheless, datasets targeting other regions of the parameter space are still required to further improve these models. We maintained consistent viewing conditions throughout the experiment. Table 2 summarizes potential biases introduced by the experimental setup and observers, as well as the measures taken to mitigate them. Despite these efforts, some residual biases may remain. These biases can be categorized into two types: biases consistent across all measurements, and biases that vary between measurements. To assess the impact of the first type, we verified that our measurements aligned with previous datasets and evaluated their consistency, as described in Section 3.1. For the second type, we confirmed that such biases were not present as the model performance was not undermined: the CDM optimized on the training subset of our measured data achieved high accuracy on the validation subset, as detailed in Section 3.2. These two independent verifications demonstrate that our dataset collection process was not significantly influenced by large biases.

### 2.3. Collected Data

We collected comprehensive human color perception threshold data, covering small and large color differences (CDs) across 136 color centers and 2702 color pairs:

- TCDs were measured in 109 color centers (2046 pairs, 17 participants);
- SCDs were measured in 18 color centers (342 pairs, 9 participants);
- LCDs were measured in 17 color centers (314 pairs, 9 participants).

**Figure 9:**
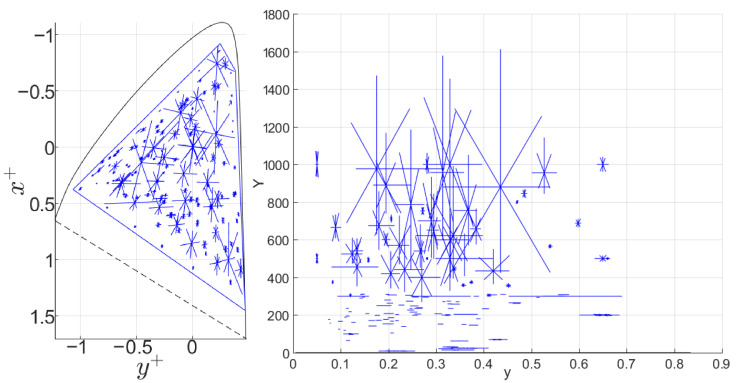
Collected data shown on the proLab chromaticity diagram (**left**) and corresponding luminance Y in cd/m^2^ (**right**). The centers of the blue segment intersections indicate the centers of the measured ellipses, while the segments themselves represent the measured color differences.

Each measurement captured three-dimensional data, forming ellipsoids in WCG and HDR areas, with color center luminances up to 1000 cd/m^2^ and a maximum luminance of 1611 cd/m^2^. Figure 9 shows the chromaticity and luminance distribution of the collected data. The convex hull of the collected color pairs forms a polygon covering 80% of the BT.2020 or BT.2100 [1] area on the proLab chromaticity diagram [36]. ProLab is a UCS based on the 3D projective transformation of CIE XYZ, with chromaticity coordinates similar to CIE xy: $x^{+}=a^{+}/L^{+}$, $y^{+}=b^{+}/L^{+}$, where $L^{+}$, $a^{+}$, and $b^{+}$ are coordinates in proLab.

The color centers were selected using two approaches. First, we measured color differences at the color centers recommended by the CIE [39] to validate our setup by comparing the results with previously published data. Second, we randomly selected color centers from a uniform distribution in proLab for luminance levels ranging from 0 to 1000 cd/m^2^. This selection excluded the sRGB color gamut (up to 300 cd/m^2^). Consequently, for the standard luminance range, we focused on highly saturated colors, while, for HDR, we explored the full saturation range.

CDs were measured in 18 directions in xyY space, including 8 in the chromaticity plane (Figure 8). In each session, the following steps were taken:

- A total of 8 pairs were measured with Δx and Δy shifts (fixed ΔY);
- If the observer was not overly fatigued, we measured 2 pairs with positive/negative ΔY (fixed Δx, Δy) and 8 pairs with combined Δx,Δy, and ΔY shifts.

A total of 231 measurement sessions were conducted, each with an observer focusing on one color center (data in certain directions were occasionally omitted if the observer reached the device’s gamut limits):

- A total of 8 directions were measured in 150 sessions (19 had partial data due to gamut limits);
- A total of 18 directions were measured in 81 sessions (3 had partial data due to gamut limits).

The order of color centers and measurement directions was randomized per observer. Each color pair within a direction was measured four times to minimize variability (following CIE recommendations [40]), using medians for analysis.

We recruited 22 observers with normal or corrected vision, who passed the Color Assessment and Diagnosis test within age-appropriate thresholds. All participants received instructions and provided informed consent.

### 2.4. Available Datasets

The widely used COMBVD dataset [41] includes small CDs (under 5 CIELAB units) and consists of four sub-datasets: BFD-P [12] with 2779 pairs, Witt [13] with 418 pairs, Leeds [14] with 307 pairs, and RIT-DuPont [15] with 312 pairs. We used this dataset to validate our own data and for CDM benchmarking. Additionally, we used the Size-dependent Color Threshold (SDCTh) dataset [42], which includes 80 pairs across 10 color centers, each measured by 10–13 observers to obtain 8 median pairs per center for stimuli with a radius of 72 min. Both datasets cover sRGB, limited to a luminance of 100 cd/m^2^ (standard dynamic range, SDR). HDR and WCG datasets exist [17,25] but are not publicly available and were thus not used here.

Table 3 summarizes the viewing conditions for each dataset (illuminant, adapting luminance $L_{a}$, luminance factor of the neutral background $Y_{b}$, and surround). Our booth lighting was set to 100 cd/m^2^, i.e., $L_{a}=100$. Since stimuli were displayed against a white background, $L_{b}=L_{w}=100$, resulting in $Y_{b}=100\left(L_{b}/L_{w}\right)=100$.

### 2.5. Quality Measures of Color Difference Models

The similarity between predicted CDs and reference measurements from human studies is commonly evaluated using the STRESS measure [43,44]. Let $\vec{x}\in R^{n}$ represent the vector of experimental CDs for *n* color pairs from a psychophysical dataset, and $\vec{y}\in R^{n}$ represent the vector of CDs for the same color pairs computed using a given CDM. The STRESS metric, $\mathrm{STRESS}(\vec{x},\vec{y})$, is defined as

(1) $$\mathrm{STRESS}\left(\vec{x},\vec{y}\right)=\frac{∥k^{*}\vec{x}-\vec{y}{∥}_{2}}{∥\vec{y}{∥}_{2}}=\sqrt{\frac{\sum_{i=1}^{n}{\left(k^{*}x_{i}-y_{i}\right)}^{2}}{\sum_{i=1}^{n}y_{i}^{2}}},$$

where $k^{*}$ is a scaling factor used for normalization:

(2) $$k^{*}=\underset{k}{\mathrm{argmin}}{∥k\vec{x}-\vec{y}∥}_{2}=\frac{{\vec{x}}^{T}\vec{y}}{{\vec{x}}^{T}\vec{x}}.$$

STRESS values range from 0 (perfect agreement) to 1 (maximum discrepancy). For context, the state-of-the-art CDM, CIEDE2000, achieves a “good” STRESS value of 0.292 on the COMBVD dataset, on which it was trained. However, this value depends on the dataset’s internal consistency and noise. The upper limit for STRESS on the new dataset is estimated in Section 3.1. In contrast, the poorly uniform CIE XYZ color space achieves a STRESS value of 0.692 on the same dataset, providing a reference for poor performance.

Various modifications have been proposed for handling multiple datasets [23,45]. Recently, a modified STRESS version, STRESS_group_, was introduced to ensure that all color pairs are accounted for with equal importance and to adjust for CD scale variations across datasets [46].

**Figure 10:**
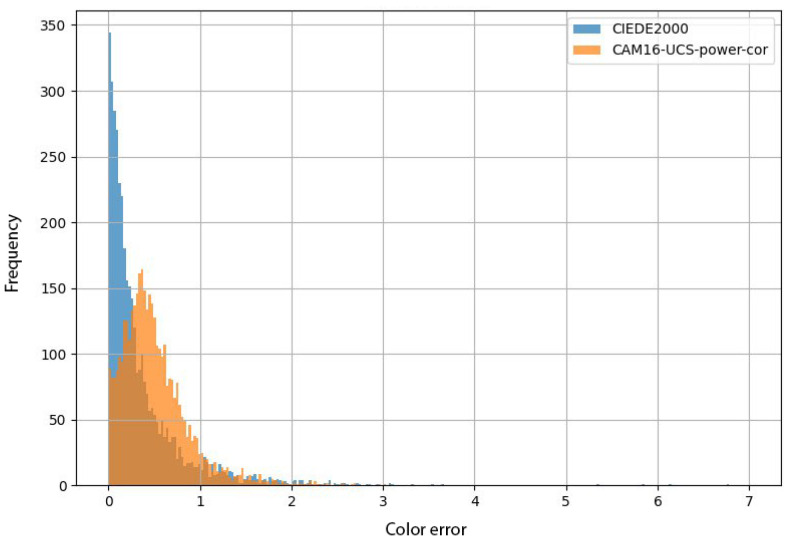
Histogram of errors for the current state-of-the-art CDMs (CIEDE2000 and CAM16-UCS-PC) on the COMBVD dataset. The x-axis represents the magnitude of the CDM error, while the y-axis indicates the number of these errors.

While STRESS evaluates the average error, it is equally important to assess significant errors, as a CDM serves as a measurement tool for evaluating other algorithms, such as those used in image processing. To address this, we propose analyzing the error distribution and focusing on errors exceeding a certain threshold, $\Delta_{0}$, which we define as unacceptable. Errors below $\Delta_{0}=1$ JND are considered imperceptible, making such a threshold unreasonable. We selected $\Delta_{0}=2$, as errors $\Delta_{0}>2$ form the heavy tail of the error distribution for current state-of-the-art CDMs (see Figure 10).

To quantify significant errors, we propose calculating the fraction of errors exceeding 2 JND:

(3) $$\mathrm{FEM}2\mathrm{JND}\left(\vec{x},\vec{y}\right)=\frac{1}{n}\sum_{i=1}^{n}\left[\left(k^{*}x_{i}-y_{i}\right)>2\right],$$

where $k^{*}$ is defined as in Equation (2).

## 3. Results

### 3.1. Comparison of the Obtained and Previously Published Data

Establishing the consistency of our experimental data with previously published data is crucial for validating our dataset. We compared our data with the COMBVD dataset [41] (see Section 2.4), which includes SCDs in the sRGB and SDR range. We focused on sub-datasets obtained under the D65 illuminant, excluding the BFD-P sub-dataset derived under the M and C illuminants.

Our consistency evaluation method leverages the fact that human color perception can be described by a continuously differentiable function without non-physiological discontinuities. This concept implies that, within a small region of color space, perceived color differences can be approximated by a linear model. By constructing a locally linear model from data in a small color space region, we can evaluate the consistency of our data with previously published datasets.

Let us consider a dataset of color pairs and their corresponding CDs. The *i*-th color pair in the dataset is represented as

(4) $$p_{i}\overset{\mathrm{def}}{=}\left(c_{i1},c_{i2}\right).$$

To define the distance between two color pairs, we first establish distances for “codirectional” and “opposite” pairs in terms of the color ordering we have chosen:

(5) $$\rho_{↑↑}\left(p_{i},p_{k}\right)=\sqrt{\frac{d\left(c_{i1},c_{k1}\right)+d\left(c_{i2},c_{k2}\right)}{2}},$$

(6) $$\rho_{↑↓}\left(p_{i},p_{k}\right)=\sqrt{\frac{d\left(c_{i1},c_{k2}\right)+d\left(c_{i2},c_{k1}\right)}{2}},$$

where *d* is a distance measure, with CIEDE2000 chosen as our metric.

The distance between color pairs is defined as

(7) $$\rho \left(p_{1},p_{2}\right)=\min\left(\rho_{↑↑}\left(p_{1},p_{2}\right),\rho_{↑↓}\left(p_{1},p_{2}\right)\right).$$

It should be noted that this distance measure may not satisfy all properties of a metric. To identify regions of high data density in color space, we constructed a complete weighted graph from the set of color pairs in the datasets, calculating all possible distances between them. We then retained only the edges with weights below a certain threshold value $\rho_{thr}$.

To identify clusters of closely positioned color pairs containing ≥N pairs, we ranked the graph’s vertices in descending order based on their degree, i.e., the number of edges connecting each vertex to its neighbors.

The following algorithm was employed for the extraction of clusters from the graph $G_{thr}$:

1. Initialize i=1.
2. If the degree of the *i*-th vertex $k_{i}>N-2$, form a cluster $C_{i}$ of size $k_{i}+1$ consisting of the *i*-th vertex and its connected vertices.
3. Remove the edges connecting each vertex in cluster $C_{i}$ to other vertices within the same cluster, and update $G_{thr}$.
4. Repeat steps 2–4 for i=i+1.

Within this cluster detection framework, any color pair can belong to multiple clusters, but any two color pairs belong to only one cluster. Sub-datasets within each cluster may vary in size.

To estimate internal consistency, we set $\rho_{thr}=2$ and N=100; then, we optimized the linear model on each cluster. Since we assumed that the units of perceptual distance may differ across datasets by a proportionality coefficient, we employed $STRESS_{group}$ [46] as the loss function.

First, we calculated the internal consistency of the COMBVD-D65 dataset as a reference. The results of the linear model optimization are presented in Table 4 for all five clusters we detected. The dataset consistency is represented by the highest value, $STRESS_{group}=0.216$.

Next, we evaluated the consistency between our data and the COMBVD-D65 dataset. The results of the linear model optimization for each detected cluster are shown in Table 5. The highest consistency value for our data with COMBVD-D65 is $STRESS_{group}=0.316$. While this is slightly worse than the internal consistency of COMBVD-D65, it remains comparable. Thus, we conclude that our data are consistent with COMBVD-D65, at least regarding the CD scale factor. We will address the issue of data consistency on an absolute scale in Section 3.3.

### 3.2. Fitting the Color Difference Model to the Data: Optimally Fitted $\Delta E_{f}$

We employed a new flexible parametric model that implements a semi-metric in color space. We also provided an analytical method for finding its optimal parameters.

Our approach built on the CAM16-UCS model with power law correction:

(8) $$\Delta E_{\mathrm{CAM}16-\mathrm{UCS}-\mathrm{PC}}=1.41{\left(\Delta E_{\mathrm{CAM}16-\mathrm{UCS}}\right)}^{0.63}.$$

We enhanced this model with an additional trainable factor for improved accuracy. The model is defined in the following linear space:

(9) $$\Delta E_{f}{\left(x,y|\theta \right)\;\overset{\mathrm{def}}{=}\;∥x-y∥}_{2}^{p}\;\left|{\left(b\left(v\left(x,y\right)\right)+b\left(v\left(y,x\right)\right)\right)}^{T}\theta \right|,$$

where $x=(J_{x},a_{x},b_{x})$, and $y=(J_{y},a_{y},b_{y})$ are CAM16-UCS coordinates of a color pair, and p=0.63. Here,

(10) $$v\left(x,y\right)\overset{\mathrm{def}}{=}\left(J_{x},\frac{\sqrt{a_{x}^{2}+b_{x}^{2}}}{J_{x}},{∥x-y∥}_{2}\right),$$

where $b:R^{3}\to R^{d}$ is a basis function dictionary listed in Appendix A, and $\theta \in R^{d}$ is the vector of CDM parameters.

This model is a semi-metric, as it does not satisfy the triangle inequality, similar to the “gold standard” CIEDE2000 formula.

To train the model, we optimized the parameter vector θ to minimize STRESS on the dataset:

(11) $$\begin{array}{c} \theta^{*}\overset{\mathrm{def}}{=}\arg\min_{\theta }\mathrm{STRESS}\left(e,M\theta \right)=\arg\min_{\theta }\min_{k}\frac{{∥ke-M\theta ∥}_{2}^{2}}{{\;\;\;∥M\theta ∥}_{2}^{2}}, \end{array}$$

where

(12) $$\begin{array}{ccc} e & \overset{\mathrm{def}}{=} & {\left[\begin{array}{cccc} e_{1} & e_{2} & \cdots & e_{n} \end{array}\right]}^{T},\\ M & \overset{\mathrm{def}}{=} & {\left[\begin{array}{cccc} m_{1} & m_{2} & \cdots & m_{n} \end{array}\right]}^{T},\\ m_{i} & \overset{\mathrm{def}}{=} & ∥x_{i}-y_{i}{∥}_{2}^{p}\;(b\left(v\left(x_{i},y_{i}\right)\right)+b\left(v\left(y_{i},x_{i}\right)\right), \end{array}$$

are vectors of experimental distances and model base matrix, respectively, and *n* represents the number of color pairs in the dataset.

This formulation results in a quadratic fractional programming problem:

(13) $$\theta^{*}\overset{\mathrm{def}}{=}\arg\min_{\theta }\min_{k}\frac{\left[\begin{array}{cc} \theta^{T} & k \end{array}\right]A{\left[\begin{array}{cc} \theta^{T} & k \end{array}\right]}^{T}}{\left[\begin{array}{cc} \theta^{T} & k \end{array}\right]B{\left[\begin{array}{cc} \theta^{T} & k \end{array}\right]}^{T}},$$

where

(14) $$A\overset{\mathrm{def}}{=}{\left[\begin{array}{cc} M & -e \end{array}\right]}^{T}\left[\begin{array}{cc} M & -e \end{array}\right],\;B\overset{\mathrm{def}}{=}{\left[\begin{array}{cc} M & 0 \end{array}\right]}^{T}\left[\begin{array}{cc} M & 0 \end{array}\right].$$

This problem can be solved analytically, avoiding numerical optimization methods. To solve (13) analytically, we first regularize matrices *A* and *B*:

(15) $$\hat{A}\overset{\mathrm{def}}{=}A+\lambda I,\;\hat{B}\overset{\mathrm{def}}{=}B+\lambda I,\;\lambda =0.01.$$

Using the Cholesky decomposition, we find an upper triangular matrix *R* such that $R^{T}R=\hat{B}$. The matrix *V* of eigenvectors of $K\overset{\mathrm{def}}{=}R^{-T}\hat{A}R^{-1}$ is

(16) $$V\overset{\mathrm{def}}{=}\left[\begin{array}{cccc} v_{1} & v_{2} & \cdots & v_{d+1} \end{array}\right]:Kv_{i}∼v_{i}.$$

The optimal solution corresponds to one of the columns of $W\overset{\mathrm{def}}{=}R^{-1}V$:

(17) $$\left[\begin{array}{c} \theta^{*}\\ k^{*} \end{array}\right]=\underset{\left[\begin{array}{c} \theta \\ k \end{array}\right]\in W}{\mathrm{argmin}}\frac{{∥ke-M\theta ∥}_{2}^{2}}{{\;\;\;∥M\theta ∥}_{2}^{2}}.$$

The optimization of CDM parameters is typically performed using numerical methods, which are computationally intensive and do not guarantee the discovery of a global minimum for non-convex objectives like STRESS. In contrast, the proposed analytical optimization approach ensures that the global minimum of STRESS is achieved while maintaining low computational complexity (approximately 0.12 s on an Intel Core i7-8550U).

The model was trained on a combined dataset comprising COMBVD, SDCTh, and our data. The dataset was split into 75% for training and 25% for testing. Using the analytical optimization Equations (15)–(17), the model achieved a STRESS value of 0.390 on the training set and 0.393 on the test set. These results demonstrate that the model is not overfitted.

### 3.3. Benchmarking Color Difference Models

We used the optimally fitted $\Delta E_{f}$ to analyze our dataset. Given the similar STRESS values across both previously published datasets and ours (see Table 6 and Table 7), we conclude that the datasets are consistent in terms of absolute CD scale. To evaluate the randomness of the obtained STRESS and FEM2JND values, we constructed confidence intervals at a confidence level of p=0.95%, corresponding to 1.96 standard deviations. For STRESS, bootstrapping [47] was applied, while the Wald confidence interval was used for FEM2JND [48].

We benchmarked the following CDMs on psychophysical datasets. CAM16-UCS, introduced in 2016 [23], is the current state-of-the-art UCS [24]. CAM16-UCS with power law correction (see Equation (8)), which accounts for the non-geodesic properties of human color space. Other UCS models designed for WCG and HDR include IC_a_C_b_ [8], IC_T_C_p_ [9], and J_z_a_z_b_z_ [10]. We also assessed Oklab [49], promising for LCD, and proLab [36], which offers good geometric properties. Additionally, we considered the CIEDE2000 formula [41], the state-of-the-art for SCD.

The results confirmed that CAM16-UCS with power correction (CAM16-UCS-PC) is the most versatile model, demonstrating the best performance on the combined dataset across both sRGB/SDR and HDR/WCG domains (see Table 8, where lower STRESS values indicate better consistency). However, CAM16-UCS-PC is not without limitations. Its accuracy could potentially improve by at least 1.3 times, as suggested by the optimally fitted $\Delta E_{f}$. For the combined dataset, the standard deviation for CAM16-UCS-PC is 0.013, while that for the optimally fitted $\Delta E_{f}$ is 0.041, meaning the difference between the mean values exceeds the standard deviation. Moreover, despite an acceptable average error, CAM16-UCS-PC shows a notable proportion of significant errors, measured as the fraction of errors above 2 JND (Table 9). A comparison between CAM16-UCS and CAM16-UCS-PC reveals that the power correction increases significant errors by 1.8 times. While CAM16-UCS-PC performs well on average, it still produces a considerable number of significant errors.

A universal model is yet to be developed, but CAM16-UCS is a promising foundation, with power correction being a key area for improvement in CAM16-UCS-PC.

## 4. Conclusions

We collected a new experimental dataset on perceived color differences spanning three scales (threshold, small, and large). The dataset covers a wide color gamut (80% of BT.2020) at high luminance levels (up to 1611 cd/m^2^) using a custom stimulus generator. Measurements were conducted with 22 observers, yielding color difference data for 2605 color pairs.

To ensure dataset consistency, we introduced a method leveraging the continuous nature of human color perception. This involved fitting linear models to small psychophysical data clusters and evaluating them using the STRESS metric. Our analysis confirmed consistency between our dataset and established datasets.

We further improved the power-corrected CAM16-UCS (CAM16-UCS-PC) model by introducing a novel color difference model, optimally fitted $\Delta E_{f}$. This refinement employed an analytical optimization approach capable of efficiently minimizing non-convex objectives, such as STRESS, while avoiding the risk of local minima. Our results demonstrated that existing models, including CAM16-UCS-PC, have not yet reached the theoretical accuracy limit, as evidenced by the performance of the optimally fitted $\Delta E_{f}$ on the combined dataset of new and established data.

Benchmarking across multiple datasets revealed that CAM16-UCS-PC is currently the most versatile model, achieving the best STRESS performance for sRGB, WCG, SDR, and HDR domains. However, the model is not without limitations. In traditional domains (SDR and sRGB), CAM16-UCS-PC exhibits fewer significant errors than CAM16-UCS. Conversely, in HDR-WCG domains, the number of significant errors for CAM16-UCS-PC is 1.8 times higher than for CAM16-UCS without power correction.

These effects can be attributed to the fact that neither model was trained on HDR or WCG data. While CAM16-UCS-PC introduces power correction to address the non-geodesic nature of color space, it remains a less appropriate model and performs worse than the base CAM16-UCS when predicting new data.

Our findings suggest that incorporating our dataset into the development of UCS could enhance future models. However, the root causes of these discrepancies remain unclear and require further investigation. Additional measurements, particularly in regions with the greatest errors, are necessary to improve model accuracy and better understand the observed effects.

## Figures and Tables

**Table 1:** Gray-scale parameters from [25] and in our experiments.

Gray-Scale Number	$L^{*}$	ΔE with GS-1	cd/m^2^ [25]	cd/m^2^, Ours
GS-1	40.0	0.0	11.2510	225.02
GS-2	41.5	1.5	12.1795	243.59
GS-3	43.0	3.0	13.1578	263.16
GS-4	46.0	6.0	15.2687	305.37
GS-5	52.0	12.0	20.1443	402.89

**Table 2:** Potential biases and the measures taken to address them.

Potential Biases	Mitigation Measures
LEDs in different quadrants may vary.	LEDs were sourced from the same model and production batch. Each quadrant was calibrated individually.
LED spectral power distribution may shift due to LED heating.	The VCD was pre-warmed before use. A fan was installed to maintain a stable temperature during operation, and the LED temperature was monitored, ensuring it remained below 40 °C.
LED spectral power distribution may shift due to room temperature fluctuations.	Room temperature was stabilized at 24 °C using air conditioning to maintain consistent LED spectral output.
The VCD may exhibit day-to-day performance variability.	Random colors were verified for accuracy using a spectrophotometer during measurements.
Room lighting may vary throughout the day.	Experiments were conducted in a fully darkened room, with D50 adaptation uniform lighting within the viewing cabin.
Observers may have color vision deficiencies.	Observers were tested using the Color Assessment and Diagnosis test, and those with color vision anomalies were excluded.
Observers may have individual variations in visual systems and color perception.	Measurements were stored for each individual separately without averaging across subjects, recognizing the importance of human diversity in perception.
Observers’ visual systems may adapt to conditions other than those in the experiment.	Observers were allowed to adapt to the experimental viewing conditions for at least 1 min before testing.
Observers may have misunderstood the task.	A learning session was conducted for each observer to discuss and clarify their observations and tasks.
Observers may adjust the number of steps arbitrarily, taking approximately the same number to find the desired color difference.	Measurement directions were randomized, and each color pair was measured four times in different directions. Medians of the measurements were used for analysis.
Observers may experience fatigue, leading to less accurate observations.	Each observer completed 2–3 sessions per day with breaks between sessions. Sessions lasted approximately 40 min, requiring several days of measurements per observer.

**Table 3:** Viewing condition parameters for experimental CD datasets.

Dataset	Illuminant	$L_{a}$	$Y_{b}$	Surround
BFD-P	D65, M, C	100	20	Average
RIT-DuPont	D65	127.3	10.9	Average
Leeds	D65	100	20	Average
Witt	D65	82.8	24.9	Average
SDCTh	D65	80	50	Average
Ours	D50	100	100	Average

**Table 4:** Results of linear model optimization for COMBVD-D65 clusters.

Region	Red	White	Blue	Yellow	Green
$STRESS_{group}$	0.197	0.179	0.181	0.216	0.197
Cluster size	294	264	206	205	202

**Table 5:** Consistency scores of COMBVD-D65 sub-datasets and our data. For each detected cluster, the number of points from each dataset (size) and the $STRESS_{group}$ metric for the correspondence of cluster points to the constructed local linear model are provided.

No.	${\mathrm{STRESS}}_{\mathrm{group}}$	BFDP Size	Rit-DuPont Size	Witt Size	Leeds Size	Our Size
1	0.240	151	6	85	52	72
2	0.260	155	18	78	13	65
3	0.316	107	—	83	15	83
4	0.242	121	—	85	—	64
5	0.237	108	—	84	9	43

**Table 6:** Performance of CDMs on previously published datasets (lower values indicate better consistency).

Model	STRESS		
	SDR & sRGB	HDR or WCG	All
CIEDE2000	0.297 ± 0.032	0.328 ± 0.077	0.306 ± 0.031
CAM16-UCS	0.303 ± 0.036	0.320 ± 0.058	0.308 ± 0.031
CAM16-UCS-PC	0.301 ± 0.014	0.293 ± 0.030	0.311 ± 0.014
J_z_a_z_b_z_	0.383 ± 0.021	0.329 ± 0.050	0.371 ± 0.021
IC_a_C_b_	0.410 ± 0.022	0.355 ± 0.050	0.398 ± 0.022
Oklab	0.475 ± 0.026	0.439 ± 0.086	0.466 ± 0.030
proLab	0.475 ± 0.024	0.439 ± 0.054	0.466 ± 0.022
IC_T_C_p_	0.492 ± 0.027	0.408 ± 0.061	0.472 ± 0.026
Opt. fitted $\Delta E_{f}$	0.334 ± 0.021	0.303 ± 0.038	0.327 ± 0.019

**Table 7:** Performance of CDMs on the dataset we collected (lower values indicate better consistency).

Model	STRESS		
	SDR & sRGB	HDR or WCG	All
CAM16-UCS-PC	0.326 ± 0.026	0.500 ± 0.033	0.528 ± 0.032
J_z_a_z_b_z_	0.470 ± 0.055	0.584 ± 0.046	0.583 ± 0.044
IC_a_C_b_	0.485 ± 0.046	0.583 ± 0.040	0.589 ± 0.037
CAM16-UCS	0.486 ± 0.033	0.625 ± 0.068	0.625 ± 0.063
Oklab	0.461 ± 0.111	0.634 ± 0.043	0.633 ± 0.043
IC_T_C_p_	0.564 ± 0.045	0.601 ± 0.038	0.635 ± 0.034
proLab	0.471 ± 0.048	0.646 ± 0.044	0.648 ± 0.042
CIEDE2000	0.525 ± 0.035	0.694 ± 0.089	0.693 ± 0.082
Opt. fitted $\Delta E_{f}$	0.439 ± 0.039	0.401 ± 0.099	0.408 ± 0.091

**Table 8:** Performance of CDMs on the combined dataset: previously published and ours (lower values indicate better consistency).

Model	STRESS		
	SDR & sRGB	HDR or WCG	All
CAM16-UCS-PC	0.395 ± 0.023	0.490 ± 0.031	0.493 ± 0.026
IC_a_C_b_	0.484 ± 0.030	0.571 ± 0.039	0.558 ± 0.033
J_z_a_z_b_z_	0.495 ± 0.035	0.584 ± 0.045	0.589 ± 0.042
CAM16-UCS	0.438 ± 0.036	0.622 ± 0.069	0.610 ± 0.064
proLab	0.508 ± 0.027	0.635 ± 0.042	0.616 ± 0.038
IC_T_C_p_	0.528 ± 0.027	0.615 ± 0.033	0.630 ± 0.027
Oklab	0.532 ± 0.037	0.641 ± 0.042	0.656 ± 0.037
CIEDE2000	0.454 ± 0.036	0.688 ± 0.090	0.668 ± 0.090
Opt. fitted $\Delta E_{f}$	0.377 ± 0.024	0.395 ± 0.096	0.393 ± 0.080

**Table 9:** Performance of CDMs on the combined dataset: previously published and ours (lower values indicate better consistency).

Model	FEM2JND		
	SDR & sRGB	HDR or WCG	All
CAM16-UCS-PC	0.026 ± 0.010	0.342 ± 0.037	0.164 ± 0.018
IC_a_C_b_	0.050 ± 0.013	0.266 ± 0.034	0.139 ± 0.017
J_z_a_z_b_z_	0.046 ± 0.013	0.250 ± 0.033	0.123 ± 0.016
CAM16-UCS	0.038 ± 0.012	0.194 ± 0.031	0.090 ± 0.014
proLab	0.063 ± 0.015	0.309 ± 0.036	0.172 ± 0.018
IC_T_C_p_	0.076 ± 0.016	0.356 ± 0.037	0.257 ± 0.021
Oklab	0.065 ± 0.015	0.246 ± 0.033	0.131 ± 0.016
CIEDE2000	0.043 ± 0.013	0.182 ± 0.030	0.095 ± 0.014
Opt. fitted $\Delta E_{f}$	0.019 ± 0.008	0.179 ± 0.030	0.081 ± 0.013

## Data Availability

The data presented in this study are available on request from the corresponding author due to the fact that the data are shared by several organizations and the issue of their transfer will be decided individually each time.

## Abbreviations

The following abbreviations are used in this manuscript:

CDM	Color difference model
CD	Color difference
UCS	Uniform color space
WCG	Wide color gamut
HDR	High dynamic range
TCD	Threshold color difference
JND	Just noticeable difference
SCD	Small color difference
LCD	Large color difference
LED	Light emitting diode
SPD	Spectral power distribution
GS	Gray scale
SDR	Standard dynamic range

## Appendix A. Basis Functions and Their Values for Optimally Fitted $\Delta E_{f}$

We selected 92 basis functions for Equation (8):

(A1) $$b={\left(b_{i}\right)}_{i=1}^{92}:$$

(A2) $$b_{1}=1,\;$$

(A3) $$\begin{array}{c} b_{2}=\sqrt{v_{1}},\\ b_{3}=\sqrt{v_{2}}, \end{array}\;\begin{array}{c} b_{4}=v_{1},\\ b_{5}=v_{2}, \end{array}\;\begin{array}{c} b_{6}=\sqrt{v_{1}v_{2}},\\ b_{7}=v_{1}v_{2}, \end{array}\;\begin{array}{c} b_{8}=v_{1}^{1.5},\\ b_{9}=v_{2}^{1.5}, \end{array}\;$$

(A4) $$\begin{array}{c} b_{10}=v_{1}^{2},\\ b_{11}=v_{2}^{2}, \end{array}\;\begin{array}{c} b_{12}=v_{1}^{3},\\ b_{13}=v_{2}^{3}, \end{array}\;\begin{array}{c} b_{14}=v_{1}^{2}v_{2},\\ b_{15}=v_{1}v_{2}^{2}, \end{array}\;\begin{array}{c} b_{16}={\left(v_{1}v_{2}\right)}^{2},\\ b_{17}={\left(v_{1}v_{2}\right)}^{3}, \end{array}\;$$

(A5) $$\begin{array}{c} b_{18}=v_{3},\\ b_{19}=v_{1}v_{3},\\ b_{20}=v_{2}v_{3}, \end{array}\;\begin{array}{c} b_{21}=v_{3}^{2},\\ b_{22}=v_{1}v_{3}^{2},\\ b_{23}=v_{2}v_{3}^{2}, \end{array}\;\begin{array}{c} b_{24}=v_{3}^{3},\\ b_{25}=v_{1}v_{3}^{3},\\ b_{26}=v_{2}v_{3}^{3}, \end{array}\;\begin{array}{c} b_{27}=v_{3}^{4},\\ b_{28}=v_{1}v_{3}^{4},\\ b_{29}=v_{2}v_{3}^{4}, \end{array}\;\begin{array}{c} b_{30}=v_{3}^{5},\\ b_{31}=v_{1}v_{3}^{5},\\ b_{32}=v_{2}v_{3}^{5}, \end{array}\;$$

(A6) $$\begin{array}{c} b_{27+6\omega }=\sin\left(2\omega v_{1}\right),\\ b_{28+6\omega }=\sin\left(2\omega v_{2}\right),\\ b_{29+6\omega }=\cos\left(2\omega v_{1}\right),\\ b_{30+6\omega }=\cos\left(2\omega v_{2}\right), \end{array}\;\begin{array}{c} b_{31+6\omega }=\sin\left(2\omega \sqrt{v_{1}v_{2}}\right),\\ b_{32+6\omega }=\cos\left(2\omega \sqrt{v_{1}v_{2}}\right), \end{array}\;\omega =1,2,\cdots ,10.$$

The values of these basis functions are shown in Table A1.

**Table A1:** Values of the basic functions.

$b_{i}$	Value	$b_{i}$	Value	$b_{i}$	Value	$b_{i}$	Value
$b_{1}$	−0.0029	$b_{24}$	2.976 × 10^−6^	$b_{47}$	−0.0002	$b_{70}$	−0.0166
$b_{2}$	−0.0057	$b_{25}$	−1.8581 × $10^{-8}$	$b_{48}$	0.0117	$b_{71}$	−2.5185 × $10^{-6}$
$b_{3}$	−0.004	$b_{26}$	3.9624 × $10^{-7}$	$b_{49}$	4.8434 × $10^{-5}$	$b_{72}$	0.028
$b_{4}$	0.0009	$b_{27}$	−4.9423 × $10^{-8}$	$b_{50}$	2.406 × $10^{-5}$	$b_{73}$	3.8023 × $10^{-5}$
$b_{5}$	0.0023	$b_{28}$	3.0614 × $10^{-10}$	$b_{51}$	3.3194 × $10^{-5}$	$b_{74}$	−7.4377 × $10^{-5}$
$b_{6}$	0.0066	$b_{29}$	−8.5736 × $10^{-9}$	$b_{52}$	0.007	$b_{75}$	−0.0003
$b_{7}$	−0.0008	$b_{30}$	2.8571 × $10^{-10}$	$b_{53}$	5.5821 × $10^{-5}$	$b_{76}$	−0.0289
$b_{8}$	−7.1602 × $10^{-5}$	$b_{31}$	−1.7522 × $10^{-12}$	$b_{54}$	−0.0036	$b_{77}$	3.0788 × $10^{-5}$
$b_{9}$	−0.0034	$b_{32}$	5.5611 × $10^{-11}$	$b_{55}$	4.657 × $10^{-5}$	$b_{78}$	−0.0304
$b_{10}$	2.2075 × $10^{-6}$	$b_{33}$	8.8041 × $10^{-5}$	$b_{56}$	−0.0001	$b_{79}$	−0.0002
$b_{11}$	−0.0035	$b_{34}$	0.0096	$b_{57}$	−0.0002	$b_{80}$	4.6816 × $10^{-5}$
$b_{12}$	−3.9465 × $10^{-10}$	$b_{35}$	−4.5735 × $10^{-6}$	$b_{58}$	0.002	$b_{81}$	−1.1926 × $10^{-5}$
$b_{13}$	−0.0084	$b_{36}$	−0.0034	$b_{59}$	4.3452 × $10^{-5}$	$b_{82}$	0.0207
$b_{14}$	−1.881 × $10^{-7}$	$b_{37}$	−8.2306 × $10^{-5}$	$b_{60}$	0.0242	$b_{83}$	3.1525 × $10^{-5}$
$b_{15}$	−4.2926 × $10^{-5}$	$b_{38}$	1.5887 × $10^{-5}$	$b_{61}$	1.9099 × $10^{-5}$	$b_{84}$	−0.0113
$b_{16}$	5.8133 × $10^{-6}$	$b_{39}$	−4.6291 × $10^{-5}$	$b_{62}$	−0.0002	$b_{85}$	−8.7425 × $10^{-5}$
$b_{17}$	−1.7872 × $10^{-8}$	$b_{40}$	0.0017	$b_{63}$	6.9818 × $10^{-5}$	$b_{86}$	1.7498 × $10^{-5}$
$b_{18}$	0.0006	$b_{41}$	−3.2055 × $10^{-5}$	$b_{64}$	−0.003	$b_{87}$	6.1974 × $10^{-5}$
$b_{19}$	−3.8521 × $10^{-6}$	$b_{42}$	-0.0089	$b_{65}$	0.0002	$b_{88}$	0.0016
$b_{20}$	4.0407 × $10^{-5}$	$b_{43}$	4.5402 × $10^{-5}$	$b_{66}$	−0.0001	$b_{89}$	−1.3631 × $10^{-5}$
$b_{21}$	−6.9548 × $10^{-5}$	$b_{44}$	9.0806 × $10^{-5}$	$b_{67}$	−0.0002	$b_{90}$	0.0055
$b_{22}$	4.3857 × $10^{-7}$	$b_{45}$	3.8185 × $10^{-7}$	$b_{68}$	−2.5034 × $10^{-5}$	$b_{91}$	4.3339 × $10^{-5}$
$b_{23}$	−5.5443 × $10^{-6}$	$b_{46}$	0.0106	$b_{69}$	9.7498 × $10^{-6}$	$b_{92}$	0.0001

## Appendix B. Formula for $J_{z}a_{z}b_{z}$

In this section, we provide the formulas for the uniform color space $J_{z}a_{z}b_{z}$ [10].

(A7) $$\left[\begin{array}{c} X^{\prime }\\ Y^{\prime } \end{array}\right]=\left[\begin{array}{c} bX\\ bY \end{array}\right]-\left[\begin{array}{c} (b-1)Z\\ (g-1)X \end{array}\right],$$

(A8) $$\left[\begin{array}{c} L\\ M\\ S \end{array}\right]=\left[\begin{array}{c} 0.41478972\;\;0.579999\;0.0146480\\ -0.2015100\;1.120649\;0.0531008\\ -0.0166008\;0.264800\;0.6684799 \end{array}\right]\left[\begin{array}{c} X^{\prime }\\ Y^{\prime }\\ Z \end{array}\right],$$

(A9) $$\left\{L^{\prime }\;M^{\prime }\;S^{\prime }\right\}={\left(\frac{c_{1}+c_{2}{\left(\frac{\left\{L\;M\;S\right\}}{10,000}\right)}^{n}}{1+c_{3}{\left(\frac{\left\{L\;M\;S\right\}}{10,000}\right)}^{n}}\right)}^{p},$$

(A10) $$\left[\begin{array}{c} I_{z}\\ a_{z}\\ b_{z} \end{array}\right]=\left[\begin{array}{ccc} 0.5 & 0.5 & 0\\ 3.524000 & -4.066708 & 0.542708\\ 0.199076 & 1.096799 & -1.295875 \end{array}\right]\left[\begin{array}{c} L^{\prime }\\ M^{\prime }\\ S^{\prime } \end{array}\right],$$

(A11) $$J_{z}=\frac{\left(1+d\right)I_{z}}{1+dIz}-d_{0},$$

where the variables *X*, *Y*, and *Z* represent the input CIE XYZ tristimulus values. $X^{\prime }$ and $Y^{\prime }$ are pre-adjusted values of *X* and *Y*, respectively, used to eliminate hue deviations before optimizing the model for improved perceptual uniformity. L, M, and *S* denote the cone primary responses, while $L^{\prime }$, $M^{\prime }$, and $S^{\prime }$ are dynamically transformed cone responses. The constants are defined as follows: b=1.15, g=0.66, $c_{1}=3424/2^{12}$, $c_{2}=2413/2^{7}$, $c_{3}=2392/2^{7}$, $n=2610/2^{14}$, $p=4289,1/2^{5}$, d=−0.56, and $d_{0}=$ 1.6295499532821566 × $10^{-11}$.

## Appendix C. Formulas for CAM16-UCS and CAM16-UCS-PC

This section outlines the formulas for CAM16-UCS and CAM16-UCS with power-law correction [23].

The initial step involves calculating values and parameters that are independent of the input sample:

(A12) $$\left[\begin{array}{c} R_{w}\\ G_{w}\\ B_{w} \end{array}\right]=M_{16}\left[\begin{array}{c} X_{w}\\ Y_{w}\\ Z_{w} \end{array}\right],\;M_{16}=\left[\begin{array}{ccc} 0.401288 & 0.650173 & -0.051461\\ -0.250268 & 1.204414 & 0.045854\\ -0.002079 & 0.048952 & 0.953127 \end{array}\right],$$

where $X_{w}$, $Y_{w}$, and $Z_{w}$ represent the adopted white in the test illuminant, and $R_{w}$, $G_{w}$, and $B_{w}$ are sensor response signals.

(A13) $$D=F\left(1-\left(\frac{1}{3.6}\right)e^{\left(\frac{-L_{A}-42}{92}\right)}\right),$$

where *D* is the degree of adaptation, $L_{A}$ is the luminance of the test adapting field (cd/m^2^), and *F* is a parameter related to the influence of the environment on color perception.

If *D* exceeds 1 or is less than 0, it is constrained to 1 or 0, respectively.

(A14) $$D_{R}=D\frac{Y_{w}}{R_{w}}+1-D,\;D_{G}=D\frac{Y_{w}}{G_{w}}+1-D,\;D_{B}=D\frac{Y_{w}}{B_{w}}+1-D,$$

(A15) $$F_{L}=0.2k^{4}\left(5L_{A}\right)+0.1{\left(1-k^{4}\right)}^{2}{\left(5L_{A}\right)}^{\frac{1}{3}},$$

where $k=\frac{1}{5L_{A}+1}$.

(A16) $$n=\frac{Y_{b}}{Y_{w}},\;z=1.48+\sqrt{n},\;N_{bb}=0.725{\left(\frac{1}{n}\right)}^{0.2},\;N_{cb}=N_{bb},$$

(A17) $$\left[\begin{array}{c} R_{wc}\\ G_{wc}\\ B_{wc} \end{array}\right]=\left[\begin{array}{c} D_{R}R_{w}\\ D_{G}G_{w}\\ D_{B}B_{w} \end{array}\right],$$

(A18) $$\left\{R_{aw}\;C_{aw}\;B_{aw}\right\}=400\left(\frac{{\left(\frac{F_{L}\left\{R_{wc}\;G_{wc}\;B_{wc}\right\}}{100}\right)}^{0.42}}{{\left(\frac{F_{L}\left\{R_{wc}\;G_{wc}\;B_{wc}\right\}}{100}\right)}^{0.42}+27.13}\right)+0.1,$$

(A19) $$A_{w}=\left(2R_{aw}+G_{aw}+\frac{B_{aw}}{20}-0.305\right)N_{bb},$$

where $A_{w}$ is the achromatic response.

Further calculation steps are as follows:

(A20) $$\left[\begin{array}{c} R\\ G\\ B \end{array}\right]=M_{16}\left[\begin{array}{c} X\\ Y\\ Z \end{array}\right],\;\left[\begin{array}{c} R_{c}\\ G_{c}\\ B_{c} \end{array}\right]=\left[\begin{array}{c} D_{R}R\\ D_{G}G\\ D_{B}B \end{array}\right],$$

(A21) $$R_{a}=400\left(\frac{{\left(\frac{F_{L}R_{c}}{100}\right)}^{0.42}}{{\left(\frac{F_{L}R_{c}}{100}\right)}^{0.42}+27.13}\right)+0.1,$$

If $R_{c}$ is negative,

(A22) $$R_{a}=-400\left(\frac{{\left(\frac{-F_{L}R_{c}}{100}\right)}^{0.42}}{{\left(\frac{-F_{L}R_{c}}{100}\right)}^{0.42}+27.13}\right)+0.1,$$

where $R_{a}$ is the postadaptation cone response, resulting in dynamic range compression. $G_{a}$ and $B_{a}$ are computed similarly.

(A23) $$a=R_{a}-\frac{12G_{a}}{11}+\frac{B_{a}}{11},\;b=\frac{\left(R_{a}+G_{a}-2B_{a}\right)}{9},\;h=tan^{-1}\left(\frac{b}{a}\right),$$

where *a* represents the Redness–Greenness component, *b* the Yellowness–Blueness component, and *h* the hue angle.

Using the unique hue data in Table A2, we set $h^{\prime }=h+360$ if $h<h_{1}$; otherwise, $h^{\prime }=h$. We choose an appropriate *i* (*i* = 1, 2, or 3) such that $h_{i}\leq h^{\prime }\leq h_{i+1}$.

(A24) $$e_{t}=\frac{1}{4}\left(cos\left(\frac{h^{\prime }\pi }{180}+2\right)+3.8\right),$$

where $e_{t}$ is the eccentricity factor.

(A25) $$H=H_{i}+\frac{100\frac{h^{\prime }-h_{i}}{e_{i}}}{\frac{h^{\prime }-h_{i}}{e_{i}}+\frac{h_{i+1}-h^{\prime }}{e_{i+1}}},$$

where *H* is the hue quadrature.

(A26) $$A=\left(2R_{a}+G_{a}+\frac{B_{a}}{20}-0.305\right)N_{bb},$$

where *A* is the achromatic response.

**Table A2:** Unique hue data for calculation of the hue quadrature.

	Red	Yellow	Green	Blue	Red
*i*	1	2	3	4	5
$h_{i}$	20.14	90.00	164.25	237.53	380.14
$e_{i}$	0.8	0.7	1.0	1.2	0.8
$H_{i}$	0.0	100.0	200.0	300.0	400.0

The correlate of lightness *J* is calculated as

(A27) $$J=100{\left(\frac{A}{A_{w}}\right)}^{cz},\;Q=\left(\frac{4}{c}\right){\left(\frac{J}{100}\right)}^{0.5}\left(A_{w}+4\right)F_{L}^{0.25},$$

where *Q* is the correlate of brightness.

The last step is the calculation of chroma (*C*), colorfulness (*M*), and saturation (*s*):

(A28) $$t=\frac{\left(\frac{50,000}{13}N_{c}N_{cb}\right)e_{t}{\left(a^{2}+b^{2}\right)}^{\frac{1}{2}}}{R_{a}+G_{a}+\left(\frac{21}{20}\right)B_{a}},$$

(A29) $$C=t^{0.9}{\left(\frac{J}{100}\right)}^{0.5}{\left(1.64-0.29^{n}\right)}^{0.73},\;M=CF_{L}^{0.25},\;s=100{\left(\frac{M}{Q}\right)}^{0.5}.$$

The CAM16-UCS is defined as

(A30) $$J^{\prime }=\frac{1.7J}{1+0.007J},\;M^{\prime }=ln\left(1+0.0228M\right)/0.0228,\;a^{\prime }=M^{\prime }cos\left(h\right),\;b^{\prime }=M^{\prime }sin\left(h\right).$$

The color difference between two colors is determined by the Euclidean distance in CAM16-UCS:

(A31) $$\Delta E_{\mathrm{CAM}16-\mathrm{UCS}}^{\prime }=\sqrt{\Delta J^{\prime 2}+\Delta a^{\prime 2}+\Delta b^{\prime 2}}.$$

The use of the power correction function can further improve CAM16-UCS:

(A32) $$\Delta E_{\mathrm{CAM}16-\mathrm{UCS}-\mathrm{PC}}=1.41{\left(\Delta E_{\mathrm{CAM}16-\mathrm{UCS}}^{\prime }\right)}^{0.63}.$$
